# Correlation between bioluminescent blinks and swimming behavior in the splitfin flashlight fish *Anomalops katoptron*

**DOI:** 10.1186/s12862-024-02283-6

**Published:** 2024-07-10

**Authors:** Peter Jägers, Timo Frischmuth, Stefan Herlitze

**Affiliations:** Department of General Zoology and Neurobiology, Institute of Biology and Biotechnology, Ruhr- University Bochum, 44801 Bochum, Germany

**Keywords:** Bioluminescence, Flashlight fish, Predator distraction, Blink and run, Animal movement, Protean behavior

## Abstract

**Background:**

The light organs of the splitfin flashlight fish *Anomalops katoptron* are necessary for schooling behavior, to determine nearest neighbor distance, and to feed on zooplankton under dim light conditions. Each behavior is coupled to context-dependent blink frequencies and can be regulated via mechanical occlusion of light organs. During shoaling in the laboratory individuals show moderate blink frequencies around 100 blinks per minute. In this study, we correlated bioluminescent blinks with the spatio-temporal dynamics of swimming profiles in three dimensions, using a stereoscopic, infrared camera system.

**Results:**

Groups of flashlight fish showed intermediate levels of polarization and distances to the group centroid. Individuals showed higher swimming speeds and curved swimming profiles during light organ occlusion. The largest changes in swimming direction occurred when darkening the light organs. Before *A. katoptron* exposed light organs again, they adapted a nearly straight movement direction.

**Conclusions:**

We conclude that a change in movement direction coupled to light organ occlusion in *A. katoptron* is an important behavioral trait in shoaling of flashlight fish.

**Supplementary Information:**

The online version contains supplementary material available at 10.1186/s12862-024-02283-6.

## Background

In fish, motion is ubiquitous and depends on numerous external (e.g. predatory pressure or environmental stress; [1]) and internal (e.g. genetic or physiological; [2]) factors. To enhance fitness in a changing environment or under threat, motion is necessary. This becomes obvious in the context-dependent movement profiles such as startle responses [3], freezing [4] or unpredictable, erratic changes of swimming direction that can help to distract predators ( [5, 6]; also see [7]).

Most fish adjust their speed and turning rates to regulate their movement direction. Turning rates are negatively correlated to swimming speed due to inertial restrictions [8]. The lateral line is essential for monitoring the hydrodynamic properties of the environment, which, for example, plays a crucial role in sensing group members while shoaling [9]. Living in shoals provides numerous benefits to fish (e.g. vigilance, reproductive success or energetic benefits) [10], but requires increased group coordination to avoid collisions. To maintain group coordination, intentional signals and/or passive cues have to be detected [11] using a range of sensory modalities including vision [12], sound [13], olfaction [14], and electrocommunication [15].

The spatial and temporal organization of fish shoals shows a strong variability from highly polarized to dispersed motion [16, 17]. Polarized movements, in which individuals are aligned, are frequently observed during fast escape responses and have been proposed to reduce the risk of predator attacks [18]. In contrast, slow moving groups are more dispersed i.e. individuals show larger distances to the group’s centroid, thereby, increasing visual fields with higher probabilities to spot threats or resources. The group’s centroid is important to quantify movement speed and/or direction, and has been used, for example, to describe predator-prey interactions under ecologically relevant settings [17, 19].

The transition from ordered to disordered motion is dependent on context [20], moving speed [21, 22] or group densities [23]. Other effects like lateralization can also be associated to collectively moving fish groups [24]. One of the interesting questions to understand shoaling behavior is how individual group members adjust their movements in relation to sensory cues, for example, to visual signals.

Visual signals, such as bioluminescence, show a high abundance in the ocean and have multiple functions e.g. to conceal the body contour via large amounts of photophores [25] or to create point-like light sources in visually restricted environments [26]. Besides offensive functions of bioluminescence (e.g. prey attraction), defensive functions such as screens, predator distraction, counterillumination or startle have been described [26, 27]. However, description of movement profiles in combination with bioluminescent flashes of nocturnal, marine organisms remain scarce. In fish, a distraction of predators has been proposed for *Gazza minuta* [28] and *Photoblepharon steinitzi* (Anomalopidae) [29]. Furthermore, bioluminescent backlighting in the Humboldt squid *Dosidicus gigas* is combined with specific locomotor behaviors and has been proposed to facilitate intraspecific communication. Additionally, these behavioral patterns, although filmed during the day, have been proposed to distract predators while being temporarily vulnerable during hunting [30]. The pattern of precisely timed bioluminescent flashes and movement profiles of the male ostracod *Photeros* (formerly *Vargula*) *annecohenae* has been linked to sexual courtship [31].

The bioluminescent, nocturnal flashlight fish *Anomalops katoptron* (Anomalopidae) inhabit the Indo Pacific and appear near the water surface in aggregations ranging from eight to several hundred individuals [32]. Characteristic for the Anomalopidae are sub-ocular light organs, which reach a length of 10% of the body size [32] and are densely packed with bioluminescent, symbiotic bacteria [33]. *A. katoptron* exhibit a downward rotation of the light-emitting surface to shield the bacteria’s continuous illumination [34]. By alternating light organ occlusion and exposure, individuals create distinct, context-dependent blink patterns, which have been shown to be involved in the localization of zooplankton [32], orientation towards conspecifics [35], and intraspecific communication [36]. Groups of *A. katoptron* can be either disordered under low stress conditions or polarized during threats [36]. The transition from shoal to school in *A. katoptron* is initiated when a small percentage of fish becomes motivated to change direction, while the rest of the school follow [35]. Schools swim with increased speeds coupled to polarized movement directions and synchronized bioluminescent blinks [36].

To gain an understanding of how bioluminescent signals relate to changes in movement, we used a stereoscopic, infrared camera system to record small shoals of *A. katoptron* in three dimensions.

## Methods

### Husbandry

Different batches of specimen of *Anomalops katoptron* (total of *n* = 20; total body length: 7.71 ± 0.08 cm) were obtained from DeJong Marinelife (Netherlands) in 2021 and 2022. Animals were maintained for several weeks before the experiments were carried out. No sexual dimorphism was reported in previous studies [37] and the group’s sex ratio was not determined in this study. Furthermore, no information on age was available.

In the laboratory, the light-dark cycle was set to 12 h–12 h with the dark period starting at 12 h pm CET. During the day, groups of *A. katoptron* dwell in caves and crevices with low light intensities [36]. Therefore, we placed different shelter in the tank and installed opaque PVC cover around it. The housing tank (120 cm x 60 cm x 60 cm; L x W x H) was equally subdivided by an opaque PVC plate. The compartments (58 cm x 58 cm x 55 cm; L x W x H) were connected via a sliding door and individuals were allowed to switch between sides. Standardized filter systems and aeration were used (see [32, 36] for details) to achieve steady water parameters (temperature: 25–27 °C; salinity: 34–36‰; NO_3_ < 20 mg/l; NO_2_ < 0.1 mg/l; PO_4_ < 0.1 mg/l). Once a day, short periods (< 30 s) of dim red light (TX 100; Coast; USA) were used to illuminate the tank and individual health was observed. Twice a day, individuals were fed ad-libitum under dark conditions with defrosted zooplankton and small amounts of minced salmon.

### Experimental procedure

For the experiments, one compartment of the tank was emptied, and the opaque sliding door was closed. The tank was illuminated with overhead infrared torches (ʎ_max_= 850 nm; IKV ACC-07, Inkovideo GmBH, Germany). Other light emitting sources were turned off or darkened. The experiments began at 2 pm (CET), two hours after the dark period started.

**Fig. 1:**
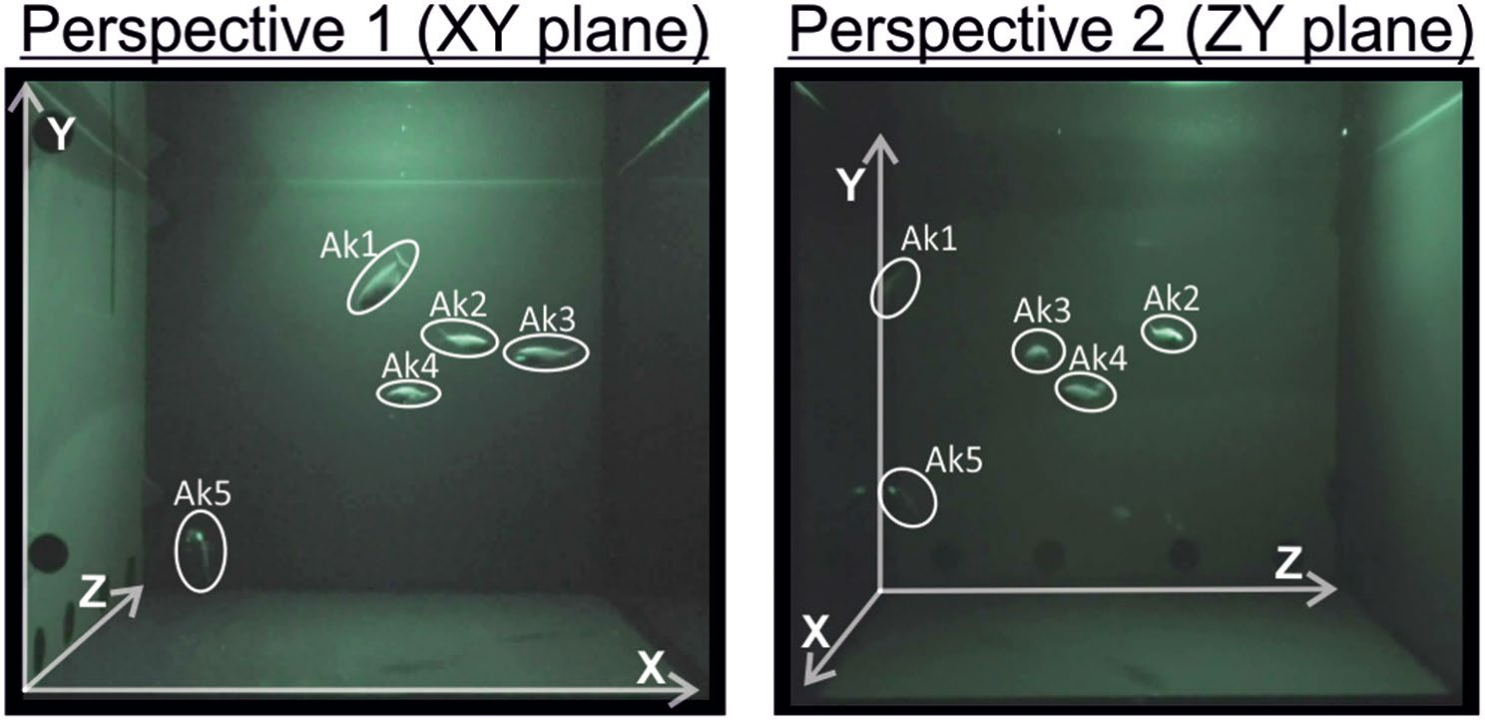
Camera Perspectives. To achieve three-dimensional tracking profiles, we used two infrared camcorder arranged in orthogonal orientation. Shown are both (XY and ZY) planes

To achieve a stereoscopic view, two, infrared-sensitive camcorder (HDR-CX730, Sony, Japan) filming with a resolution of 1920 × 1280 pixel at 25 fps were placed in an orthogonal orientation in front and at the side of the compartment (Fig. 1 and S1A). Each camcorder was placed on a tripod at the same level (160 cm in our setup) and 77 cm from the tank, matching the center of the compartment. We used the Stereo Camera Calibration toolbox of Matlab (Matlab 2022b; The MathWorks Inc., USA) to compute camera parameter by taking various photographs (*n* = 16) of a checkerboard (7 × 9; squares: 30 × 30 mm; Fig. S1A) in different orientations. The mean projection error was 0.71 pixel (Fig. S1B). The toolbox allowed us to determine intrinsic parameters for both cameras, as well as the rotation and translation of camera two in reference to camera one, which was designated as the scene’s center. Further, we calculated projection matrices for both cameras (Additional File 1; Fig. S1C).

Prior to the experiment, visibility of the tank was checked through the ocular, which then was darkened with black sheets to further reduce the bright shining IR-image emitted through the ocular of the camera. For the experiment five individuals were randomly assigned to a group. We used group sizes of five individuals with intact light organs to obtain comparability with our previous studies where we also used group sizes of five individuals (see [32, 36]). The first group was measured on the 21st of June 2021, the second group on the 31st of August 2021, and groups three and four on the 7th of September 2022. Each group was transferred with a small hand net to the measurement compartment and habituated for five minutes in total darkness. Camcorder were started and recordings took place for five minutes. After recording, individuals were transferred back into the housing compartment.

### Data analysis and statistics

We back-synchronized camcorder with brief (< 1 s), dim, red light pulses presented after each trial. Additionally, we checked multiple frames in which light organs were visible in both perspectives and controlled whether synchronization of camcorders occurred. Videos were converted to .avi-format and edited by using Shotcut (GNU General Public License; Meltytech, LLC). Two minutes ( ≙ 3000 frames) of swim and blink profiles of twenty *A. katoptron* were manually analyzed (total of 4163 blink events), frame by frame in Vidana (Vidana 2.0, Germany). The pixel coordinates (x, y) were obtained from both cameras (Fig. 1). We used a script originally written by Lourakis [38] to triangulate real world coordinates (given in mm). Here, we used the midpoint method [39] to generate projection matrices (Fig. S1). The real-world coordinates were subsequently assembled to respective time frames in Excel (Office Professional Plus 2019; Microsoft, USA). Light organ exposure (assigned to 1) and occlusion (assigned to 0) were documented and added to the excel files as soon as one light organ was exposed (see Additional File 2). Additionally, we calculated which luminous organs of the individuals were visible from each perspective in the first minute of filming. This analysis differentiated between (a) both luminous organs being visible in either one or a combination of both camera perspectives (44.66%), (b) one luminous organ being visible in at least one perspective (54.52%), or (c) no luminous organ being visible (0.81%; see Additional File 1 Fig. S3A). The estimate of simultaneous blinks was determined for the frames in which both light organs were visible. We analyzed whether both light organs were simultaneously exposed/occluded, a delay between both light organs occurred or one light organ was presented independently (see Additional File 1 Fig. S3B).

**Fig. 2:**
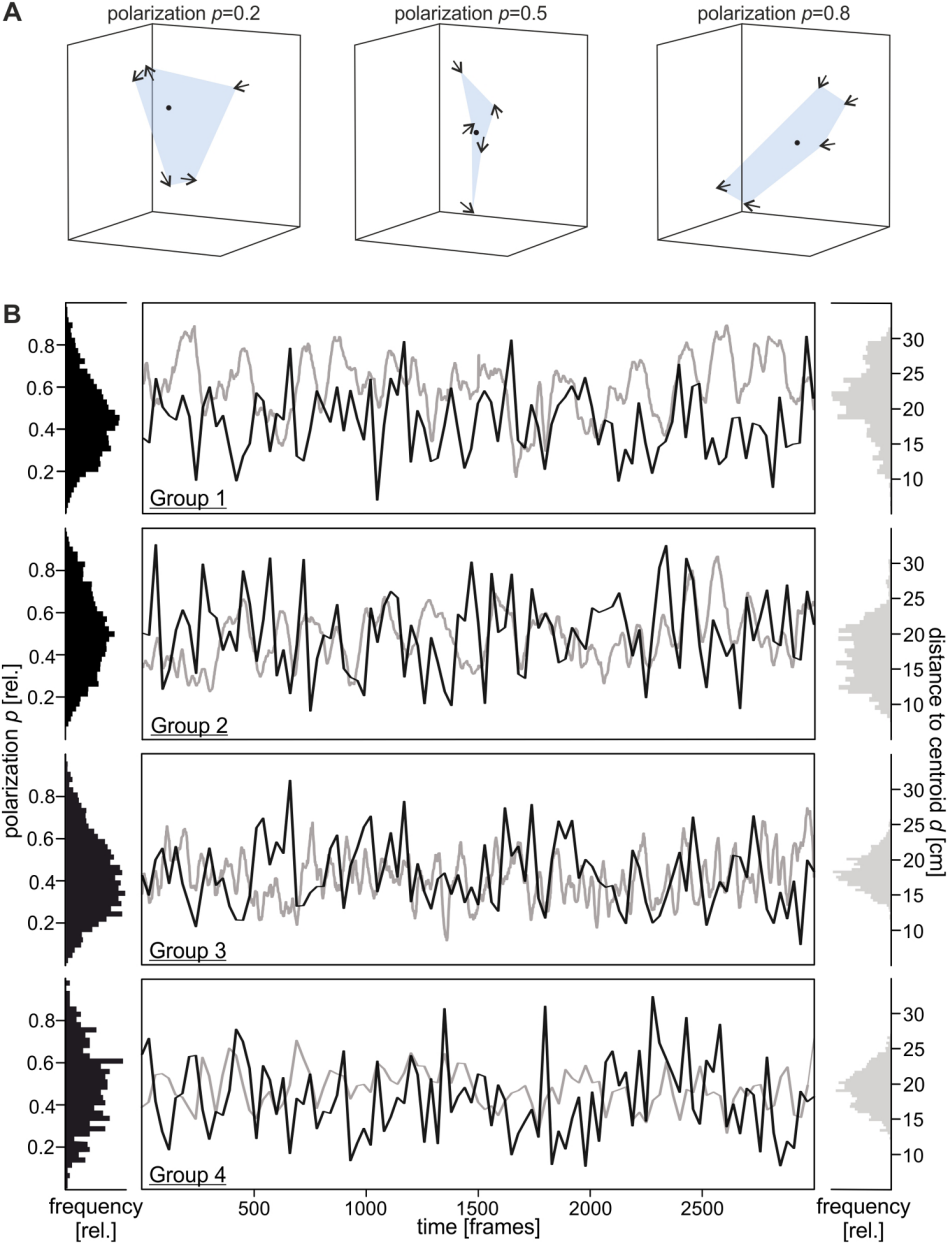
Shoaling of *A. katoptron*. Schematic representation of different levels of polarization of groups of *A. katoptron*. Arrows indicate movement directions of the individuals and the black dot the group’s centroid (**A**). Trajectories of four groups, each consisting of five individuals, were obtained from two camera perspectives. Polarization (*p*, black) and distance to the group’s centroid (*d*, grey) was characterized for 3000 frames (**B**). Histograms (bin size 40) show densities of polarization and distance to centroid for the full recording time (3000 frames). Polarization was smoothed via averaging the values at neighboring points (sampling proportion 0.001 ≙ 3 frames; fraction of a total number of data points used to compute each smoothed value)

To explain group features, we examined individual distances to the group’s centroid and polarization (Fig. 2), which offers a measure of alignment of individuals inside the group. The value of the individual (*n*) heading at time point *t*, where *u*_*i*_ is the unit vector of fish number *i*, was used to derive polarization (*p*, Eq. 1).

1 $$p \left(t\right)= \frac{1}{n} \left|{\sum }_{i=1}^{n}{u}_{i} \left(t\right)\right|$$

Values reached *p* = 1 when all individuals were aligned, whereas *p* = 0 when no alignment existed. The group’s centroid was determined as the mean of all individual coordinates of the group at timestep *t*. Respectively, distance to the group’s centroid (*d*) was calculated and averaged for all individuals within the group. Speed and change of swimming direction were calculated with self-written Matlab programs (Matlab 2020a; The MathWorks, Inc., USA). Thereby, instantaneous speed *s*(*t*) was the distances between coordinates at time *t* (Eq. 2).

2 $$s\left(t\right)= \frac{\sqrt{{(x\left(t\right)-x(t-1\left)\right)}^{2}+ {(y\left(t\right)-y(t-1\left)\right)}^{2}+{(z\left(t\right)-z(t-1\left)\right)}^{2}}}{dt}$$

Here, *dt* is the length of the time interval (*dt* = 1/fps ≙ 0.04 s) and *x(t)*, *y(t)* and *z(t)* are the *x*, *y* and *z* coordinates of one fish at time *t*. The change in direction (α) was calculated by the arccosine of two vectors (Eq. 3). Each vector (*u, v*) was determined for a pair of coordinates, vector *u* at timepoints (t), (t - 1) and *v* at timepoints (t), (t + 1). A 180-degree angle is equal to a U-turn, whereas 0-degree represents a straight line.

3 $$\alpha \;(t)\; = \;co{s^{ - 1}}\left( {\frac{{u\; \cdot \;v}}{{\left| u \right|\; \cdot \;\left| v \right|}}} \right)$$

**Fig. 3:**
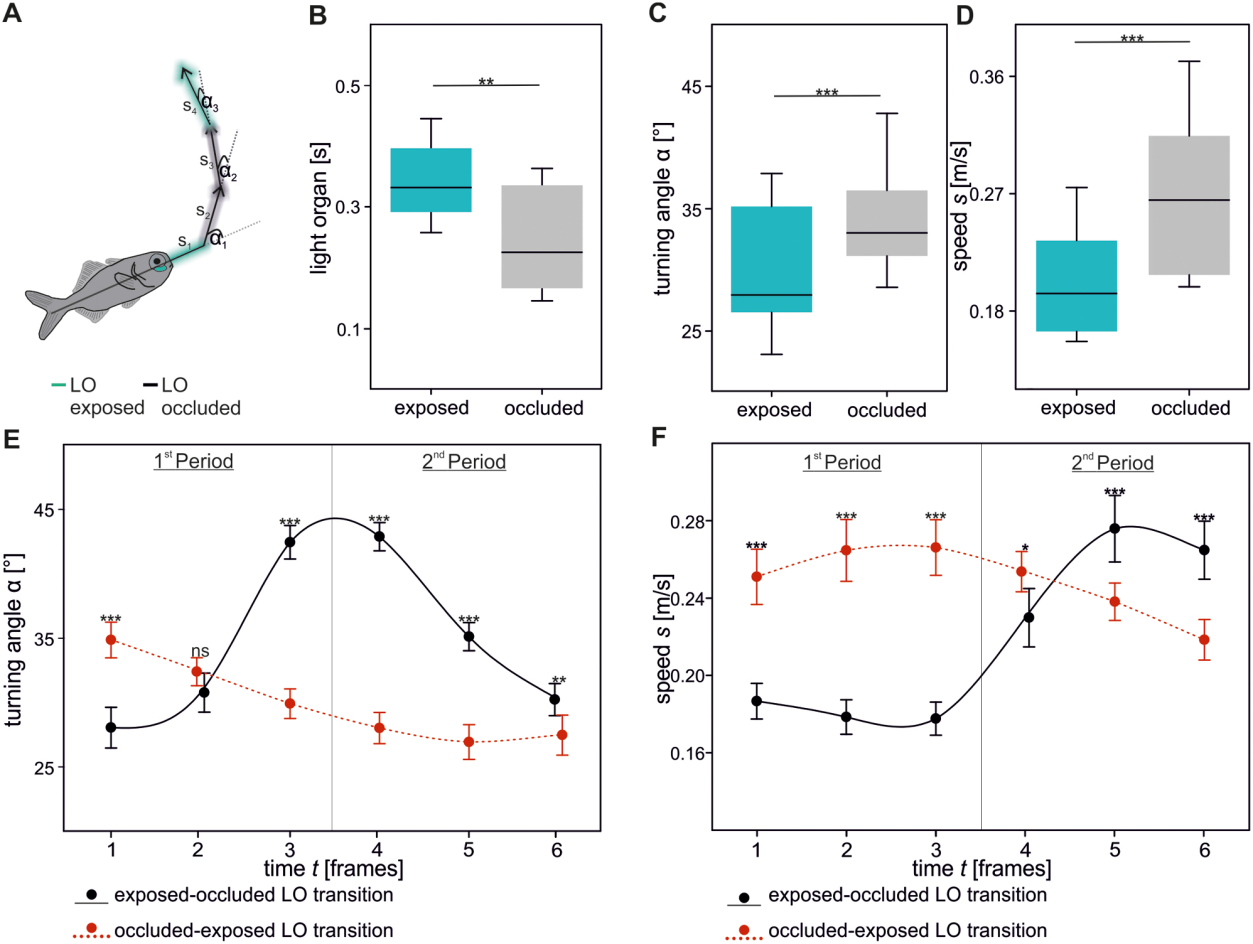
Individual movement direction in relation to light organ (LO) exposure and occlusion. Example trajectory of one individual with exposed (blue) and occluded (grey) light organs (**A**). Light organ exposure and occlusion of all tested individuals (*n* = 20) during shoaling (**B**). Differences of swimming direction (**C**) and speed (**D**) under both conditions (LO exposed or occluded). Detailed changes of swimming direction (**E**) and speed (**F**) three frames before (1–3) and after (4–6) the light organ transition. Data in B to F was obtained from twenty individuals (*n =* 20). A dynamic fitting with a polynomial, cubic equation was used to additionally plot data in E and F. Significance values are reported as *p < 0.05, **p < 0.01, ***p < 0.001. Data in E and F indicate mean ± SEM

We analyzed speed and angular changes three frames before and after phase transition of light organ exposure to occlusion and vice versa. For every individual, we calculated the mean values at each time step. In Fig. 3E and F a dynamic fitting with a polynomial, cubic equation was used to plot data (included function of SigmaPlot; version 12.0; Systat, India).

The descriptive statistic (e.g. mean and standard deviation) was calculated in Excel. The data points of all individuals were pooled and analyzed in SigmaPlot. After successful evaluation of normal distribution (Shapiro-Wilk test), differences in exposure and occlusion of light organs (Fig. 3B), and angular changes (Fig. 3C) were analyzed with a paired t-test. In case of non-normally distributed data (swimming speed; Fig. 3D), the Wilcoxon signed rank test was used to assess differences. Differences of turning angles (Fig. 3E) and swimming speeds (Fig. 3F) at the specific timesteps were evaluated via a two-way repeated measurement (rm) ANOVA and Holm-Sidak post hoc analysis. For the statistical analysis with a two-way rm ANOVA, we tested whether the data points met the assumptions that no outliers existed, were normally distributed, and sphericity was given. The analysis was performed using timestep and type of transition (either exposed to occluded light organs or vice versa) as factors (see Table S1 and S2 for detailed values). All values are reported as mean ± SEM (standard error of mean). Significant differences are reported as: * *p* ≤ 0.05, ** *p* ≤ 0.01; *** *p* ≤ 0.001.

## Figures

Figures were created with SigmaPlot 12.0 (www.sigmaplot.co.uk) and Matlab (Matlab 2022b; The MathWorks Inc., USA), and processed with CorelDraw Graphics Suite 2017 (www.coreldraw.com).

## Results

To investigate the correlation of the light organ occlusion/exposure with the movement profiles of the nocturnal flashlight fish *Anomalops katoptron*, we recorded three dimensional trajectories of small groups of *A. katoptron* under infrared settings (Fig. 1 and Additional File 3). To determine attributes of shoaling, we analyzed polarization, the alignment of individuals, and mean distance to the group’s centroid (Fig. 2A). Individuals within a group of *A. katoptron* did neither move in the same (values 1) nor opposite (values 0) direction (Fig. 2B). Additionally, we discovered that the distance of *A. katoptron* to the centroid occurred in a wave-like pattern and the mean polarization was moderate (Fig. 2B).

In our study, the mean light organ exposure (345 ± 14.7 ms) is different from the occlusion (245 ± 18.3 ms; *t*(19) = 3.489, *p* = 0.002; Fig. 3B). The alternating exposure and occlusion resulted in blink frequencies of 103.82 ± 4.15 blinks/min (see Additional File 2). Most of the time, the left and right light organ were exposed and occluded simultaneously (86.46%). To a smaller extent, one light organ was occluded or exposed before the other (delayed;7.97%) or one light organ was exposed independently (5.56%; see Figure S3B).

Individuals showed larger turning angles (34.31 ± 1.04 °; *t*(19) = − 7.94, *p* ≤ 0.001; Fig. 3C) and increased swimming speeds (0.267 ± 0.014 m/s; Wilcoxon signed rank: Z = 3.92, *p* ≤ 0.001; Fig. 3D) with occluded compared to exposed light organs. A more detailed analysis revealed the differences in swimming direction and speed between the transition from exposed to occluded light organs and vice versa (Fig. 3E and F). The strongest alteration became obvious when light organs were darkened. Immediately before occluding their light organs, individuals slowed down to 0.178 ± 0.008 m/s. This was combined with a change in swimming direction around the transition (42.447 ± 1.3 °, frame 3 and 42.879 ± 1.1 °, frame 4). In the consecutive frames, individuals increased swimming speed (0.265 ± 0.015 m/s, frame 6) and decreased swimming angle to 30.225 ± 1.24 ° (frame 6).

The transition from occluded to exposed light organs showed smaller alterations, indicating a continuous, straight-lined swimming profile. The swimming speed was slightly increased to approx. 0.25 m/s during the transition (frame three and four). Turning angle was nearly constant with sustained light organ exposure in frame five (26.93 ± 1.347 °) and six (27.469 ± 1.553 °).

Frame related transitions of occluded to exposed and exposed to occluded light organ were significantly different regarding turning angle (Fig. 3E; F _5, 95_ = 63.61, *p* ≤ 0.001; Table S1) and swimming speed (Fig. 3F; F _5, 95_ = 38.72, *p* ≤ 0.001; Table S2). The post-hoc analysis revealed that besides frame two for turning angle (Holm-Sidak: *p* = 0.117), all other results were highly significant (Holm-Sidak: *p* ≤ 0.001).

In summary, flashlight fish *A. katoptron* coordinate bioluminescent blinks with changes in movement profiles while shoaling.

## Discussion

In the present study, we observed that *Anomalops katoptron’*s blink and movement profiles followed a precisely timed pattern while shoaling. When individuals occluded their light organs, their swimming speed increased, and swimming direction was changed. We found that small groups of *A. katoptron* showed mean blink frequencies of 103 blinks/min with slightly increased light organ exposure compared to occlusion. Occasionally, we observed that light organs of an individual were not exposed simultaneously.

Bioluminescent signaling can enhance intraspecific communication in visually restricted environments as shown in ostracods (e.g. Cypridinidae) [31, 40], cephalopods (e.g. Ommastrephidae) [30], and fish (e.g. Leiognathidae) [28]. For group living *A. katoptron*, bioluminescent displays have been proposed to attract conspecifics [35], determine nearest neighbor distance [36], and illuminate prey [32]. Other than *A. katoptron*, individuals of the closely related genus *Photoblepharon* occur solitary or in pairs. Here, the distraction of predators via “blink and run”-pattern [29], aggression during territorial defense, and illumination of prey [41] have been linked to bioluminescent displays. Besides the benefits of bioluminescent signaling, light sources also build a strong contrast against a dark background (e.g. with increasing water depth), becoming increasingly visible to predators with appropriate visual systems [42]. To balance the visual information for conspecifics while reducing the risk of being exploited by predators is crucial.

Our study revealed that after light organs were occluded, individuals immediately changed direction and increased swimming speed. This behavioral phenomenon allows coverage for the last visual cue’s spatial position and may provide sensory confusion for potential predators. The confusion of predators either via startle or jamming its visual system has been proposed for bioluminescent fish such as *Gazza minuta* [28] and *Leiognathus elongatus* [43] (note taxonomic revision [44]). In *G. minuta* a single, bright flash was followed by darting away [28]. A similar behavior was described by Morin (1975) for *Photoblepharon steinitzi* (Anomalopidae), which typically inhabit reef caves. Here, multiple, erratic changes of swimming direction were coupled to bioluminescent flashes, a behavior described as “blink and run” pattern. Although originally describe as a defensive mechanism against predators in its reef caves or while swimming in unprotected areas, our study on *P. steinitzi* in the Red Sea revealed that a similar behavior is shown against intraspecific intruders [41]. Our results agree with the “blink and run”- hypothesis, where evasive swimming and bioluminescent blinks are coordinated [29]. For other, non-bioluminescent fish species it has been suggested that a rapid change of movement direction increases survival during predator attacks. For example, virtual prey with straight swimming trajectories (Lévy motion) were targeted more frequently by predators [45]. In addition, several other defensive functions of bioluminescence e.g. burglar alarm, distractive body parts or smoke screens have been discussed in many other species [26].

Besides the suggestion of a distractive signal, visual cues can also be important for group coordination. It has been shown that bioluminescent signals of *A. katoptron* are necessary to school under dim light conditions and small numbers of individuals can initiate changes in movement directions [35]. While schooling, for example during fast escape responses, individuals are synchronized in their movement and blinking pattern [35, 36]. Swimming speed has been positively correlated with higher group polarization in other species [46]. Furthermore, due to inertial limitations, turning rates decrease at faster swimming speeds [8]. In contrast to coordinated group behavior, our results show low polarization and swimming speed of groups of *A. katoptron* while shoaling in the tank. It has been emphasized that a correlation between group size and polarization exists. Although this might be applicable in some species ( [47]; but also note [48, 49]), flashlight fish *A. katoptron* also showed shoaling behavior in larger aggregations in the field [36]. Our data is limited to one group size and field recordings are restricted to local interactions. In addition, the tendency to shoal increases with prolonged habituation time [48]. Therefore, it might be interesting to test different group sizes under controlled conditions at different time points and how these parameters affect the transition from shoaling to schooling.

Limitations due to the spatial constraints of the tank walls which may affect the acceleration and turning response of the individuals are possible. Other species maintain a minimum distance of 5 cm towards the wall [50]. Conversely, we observed that *A. katoptron* sometimes moved in front of the mirrored tank wall in response to their own reflection (see Additional File 3). The perception of an attracting signal is most likely (similar to [36]; also note self-recognition in other species [51]).

Our manuscript does not explore the trade-off between distraction of predators and attraction of conspecifics. This needs to be addressed in future work, focusing on the information transfer during the transition from loosely organized to highly synchronized schools. Here, tracking software based on high-resolution, deep-learning approaches [52] and advanced technological approaches within field sites would be necessary [53]. Information transfer during transitions have been observed either in non-bioluminescent fish species e.g. *Notemigonus crysoleucas* [54] or terrestrial environments e.g. fireflies *Photinus carolinus* [55].

## Conclusion

In summary, our results show that individuals of *A. katoptron* correlate directional changes and light organ occlusion during shoaling.

### Electronic supplementary material

Below is the link to the electronic supplementary material.

Supplementary Material 1

Supplementary Material 2

Supplementary Material 3

## Data Availability

The datasets supporting the conclusions of this article are included within the article (and its additional files).

## References

[1] Herbert-Read JE, Rosén E, Szorkovszky A, Ioannou CC, Rogell B, Perna A (2017). How predation shapes the social interaction rules of shoaling fish. Proc Biol Sci.

[2] Killen SS, Marras S, Nadler L, Domenici P (2017). The role of physiological traits in assortment among and within fish shoals. Philos Trans R Soc Lond B Biol Sci.

[3] Domenici P, Blake R (1997). The kinematics and performance of fish fast-start swimming. J Exp Biol.

[4] Swanbrow Becker LJ, Gabor CR (2012). Effects of Turbidity and Visual vs. Chemical cues on Anti-predator Response in the endangered Fountain Darter (Etheostoma fonticola). Ethology.

[5] Humphries DA, Driver PM (1967). Erratic display as a device against predators. Science.

[6] Nair A, Changsing K, Stewart WJ, McHenry MJ (2017). Fish prey change strategy with the direction of a threat. Proc Royal Soc B: Biol Sci.

[7] Szopa-Comley AW, Ioannou CC (2022). Responsive robotic prey reveal how predators adapt to predictability in escape tactics. Proc Natl Acad Sci U S A.

[8] Klamser PP, Gómez-Nava L, Landgraf T, Jolles JW, Bierbach D, Romanczuk P (2021). Impact of variable speed on collective Movement of Animal Groups. Front Phys.

[9] Kasumyan AO (2003). The lateral line in fish: structure, function, and role in behavior. J Ichthyol.

[10] Krause J, Ruxton GD (2002). Living in groups.

[11] Dall SR, Giraldeau L-A, Olsson O, McNamara JM, Stephens DW (2005). Information and its use by animals in evolutionary ecology. Trends Ecol Evol.

[12] Kowalko JE, Rohner N, Rompani SB, Peterson BK, Linden TA, Yoshizawa M (2013). Loss of Schooling Behavior in Cavefish through Sight-Dependent and Sight-Independent mechanisms. Curr Biol.

[13] Amorim MCP, Simões JM, Almada VC, Fonseca PJ (2011). Stereotypy and variation of the mating call in the lusitanian toadfish, Halobatrachus didactylus. Behav Ecol Sociobiol.

[14] Ward AJ, Mehner T (2010). Multimodal mixed messages: the use of multiple cues allows greater accuracy in social recognition and predator detection decisions in the mosquitofish, Gambusia holbrooki. Behav Ecol.

[15] Worm M, Landgraf T, Prume J, Nguyen H, Kirschbaum F, von Emde G (2018). Der. Evidence for mutual allocation of social attention through interactive signaling in a mormyrid weakly electric fish. Proc Natl Acad Sci U S A.

[16] Katz Y, Tunstrøm K, Ioannou CC, Huepe C, Couzin ID (2011). Inferring the structure and dynamics of interactions in schooling fish. Proc Natl Acad Sci U S A.

[17] Romenskyy M, Herbert-Read JE, Ioannou CC, Szorkovszky A, Ward AJW, Sumpter DJT (2020). Quantifying the structure and dynamics of fish shoals under predation threat in three dimensions. Behav Ecol.

[18] Ioannou CC, Guttal V, Couzin ID (2012). Predatory fish select for coordinated collective motion in virtual prey. Science.

[19] Jolles JW, Boogert NJ, Sridhar VH, Couzin ID, Manica A (2017). Consistent Individual Differences Drive Collective Behavior and Group Functioning of Schooling Fish. Curr Biol.

[20] Schaerf TM, Dillingham PW, Ward AJW (2017). The effects of external cues on individual and collective behavior of shoaling fish. Sci Adv.

[21] Kent MIA, Lukeman R, Lizier JT, Ward AJW (2019). Speed-mediated properties of schooling. R Soc Open Sci.

[22] Jolles JW, Weimar N, Landgraf T, Romanczuk P, Krause J, Bierbach D (2020). Group-level patterns emerge from individual speed as revealed by an extremely social robotic fish. Biol Lett.

[23] Makris NC, Ratilal P, Jagannathan S, Gong Z, Andrews M, Bertsatos I (2009). Critical population density triggers rapid formation of vast oceanic fish shoals. Science.

[24] Bisazza A, Dadda M (2005). Enhanced schooling performance in lateralized fishes. Proc Biol Sci.

[25] Claes JM, Nilsson D-E, Straube N, Collin SP, Mallefet J (2014). Iso-Luminance counterillumination drove bioluminescent shark radiation. Sci Rep.

[26] Haddock SHD, Moline MA, Case JF (2010). Bioluminescence in the sea. Ann Rev Mar Sci.

[27] Widder EA (2010). Bioluminescence in the ocean: origins of biological, chemical, and ecological diversity. Science.

[28] McFall-Ngai MJ, Dunlap PV (1983). Three new modes of luminescence in the leiognathid fish Gazza minuta: discrete projected luminescence, ventral body flash, and buccal luminescence. Mar Biol.

[29] Morin JG, Harrington A, Nealson K, Krieger N, Baldwin TO, Hastings JW (1975). Light for all reasons: versatility in the behavioral repertoire of the Flashlight Fish. Science.

[30] Burford BP, Robison BH (2020). Bioluminescent backlighting illuminates the complex visual signals of a social squid in the deep sea. Proc Natl Acad Sci U S A.

[31] Rivers TJ, Morin JG (2008). Complex sexual courtship displays by luminescent male marine ostracods. J Exp Biol.

[32] Hellinger J, Jägers P, Donner M, Sutt F, Mark MD, Senen B (2017). The Flashlight Fish Anomalops katoptron uses bioluminescent light to Detect Prey in the Dark. PLoS ONE.

[33] Haneda Y, Tsuji FI (1971). Light production in the luminous fishes Photoblepharon and Anomalops from the Banda Islands. Science.

[34] Johnson GD, Rosenblatt RH (1988). Mechanisms of light organ occlusion in flashlight fishes, family Anomalopidae (Teleostei: Beryciformes), and the evolution of the group. Zool J Linn Soc.

[35] Gruber DF, Phillips BT, O’Brien R, Boominathan V, Veeraraghavan A, Vasan G (2019). Bioluminescent flashes drive nighttime schooling behavior and synchronized swimming dynamics in flashlight fish. PLoS ONE.

[36] Jägers P, Wagner L, Schütz R, Mucke M, Senen B, Limmon V (2021). Social signaling via bioluminescent blinks determines nearest neighbor distance in schools of flashlight fish Anomalops katoptron. Sci Rep.

[37] Steche O. Die Leuchtorgane Von Anomalops katoptron und Photoblepharon palpebratus, Zwei Oberflächenfischen aus dem Malaiischen Archipel. Ein Beitrag Zur Morphologie Und Physiologie Der Leuchtorgane Der Fische. Z für Wissenschaftliche Zool. 1909:349–408.

[38] Lourakis M, Stereo. triangulation. 2023. https://www.mathworks.com/matlabcentral/fileexchange/67383-stereo-triangulation.

[39] Hartley R, Zisserman A (2018). Multiple view geometry in computer vision.

[40] Hensley, Rivers, Gerrish, Saha R, Oakley (2023). Collective synchrony of mating signals modulated by ecological cues and social signals in bioluminescent sea fireflies. Proc Royal Soc B: Biol Sci.

[41] Hellinger J, Jägers P, Spoida K, Weiss LC, Mark MD, Herlitze S (2020). Analysis of the territorial aggressive behavior of the Bioluminescent Flashlight Fish Photoblepharon steinitzi in the Red Sea. Front Mar Sci.

[42] Warrant EJ, Locket NA (2004). Vision in the deep sea. Biol Rev Camb Philos Soc.

[43] Sasaki A, Ikejima K, Aoki S, Azuma N, Kashimura N, Wada M (2003). Field evidence for Bioluminescent Signaling in the Pony Fish, Leiognathus elongatus. Environ Biol Fish.

[44] Suzuki H, Kimura S (2017). Taxonomic revision of the equulites Elongatus (Günther 1874) species group (Perciformes: Leiognathidae) with the description of a new species. Ichthyol Res.

[45] Ioannou CC, Carvalho LAB, Budleigh C, Ruxton GD (2023). Virtual prey with Lévy motion are preferentially attacked by predatory fish. Behav Ecol.

[46] Gautrais J, Ginelli F, Fournier R, Blanco S, Soria M, Chaté H, Theraulaz G (2012). Deciphering interactions in moving animal groups. PLoS Comput Biol.

[47] Becco C, Vandewalle N, Delcourt J, Poncin P (2006). Experimental evidences of a structural and dynamical transition in fish school. Physica A.

[48] Miller N, Gerlai R (2012). From schooling to shoaling: patterns of collective motion in zebrafish (Danio rerio). PLoS ONE.

[49] Gimeno E, Quera V, Beltran FS, Dolado R (2016). Differences in shoaling behavior in two species of freshwater fish (Danio rerio and Hyphessobrycon herbertaxelrodi). J Comp Psychol.

[50] Herbert-Read JE, Perna A, Mann RP, Schaerf TM, Sumpter DJT, Ward AJW (2011). Inferring the rules of interaction of shoaling fish. Proc Natl Acad Sci U S A.

[51] Kohda M, Bshary R, Kubo N, Awata S, Sowersby W, Kawasaka K (2023). Cleaner fish recognize self in a mirror via self-face recognition like humans. Proc Natl Acad Sci U S A.

[52] Francisco FA, Nührenberg P, Jordan A (2020). High-resolution, non-invasive animal tracking and reconstruction of local environment in aquatic ecosystems. Mov Ecol.

[53] Sarfati R, Hayes JC, Sarfati É, Peleg O (2020). Spatio-temporal reconstruction of emergent flash synchronization in firefly swarms via stereoscopic 360-degree cameras. J R Soc Interface.

[54] Tunstrøm K, Katz Y, Ioannou CC, Huepe C, Lutz MJ, Couzin ID (2013). Collective states, multistability and transitional behavior in schooling fish. PLoS Comput Biol.

[55] Sarfati R, Joshi K, Martin O, Hayes JC, Iyer-Biswas S, Peleg O (2023). Emergent periodicity in the collective synchronous flashing of fireflies. Elife.

