# Supplementary material for: Correlation between bioluminescent blinks and swimming behavior in the splitfin flashlight fish Anomalops katoptron
Source: BMC Ecol Evol. 2024 Jul 10;24:97. doi: 10.1186/s12862-024-02283-6 (PMC11234731; doi:10.1186/s12862-024-02283-6)
Supplement: Supplementary file 2 — Supplementary Material 2 [file 12862_2024_2283_MOESM2_ESM.docx]

**Supplementary for the Manuscript:**

**Correlation between bioluminescent blinks and swimming behavior in the splitfin flashlight fish *Anomalops katoptron***

Peter Jägers ^1^ *, Timo Frischmuth ^1^ & Stefan Herlitze ^1^

^1^ Department of General Zoology and Neurobiology, Institute of Biology and Biotechnology, Ruhr-University Bochum, 44801 Bochum, Germany

* corresponding author: peter.jaegers@ruhr-uni-bochum.de

**
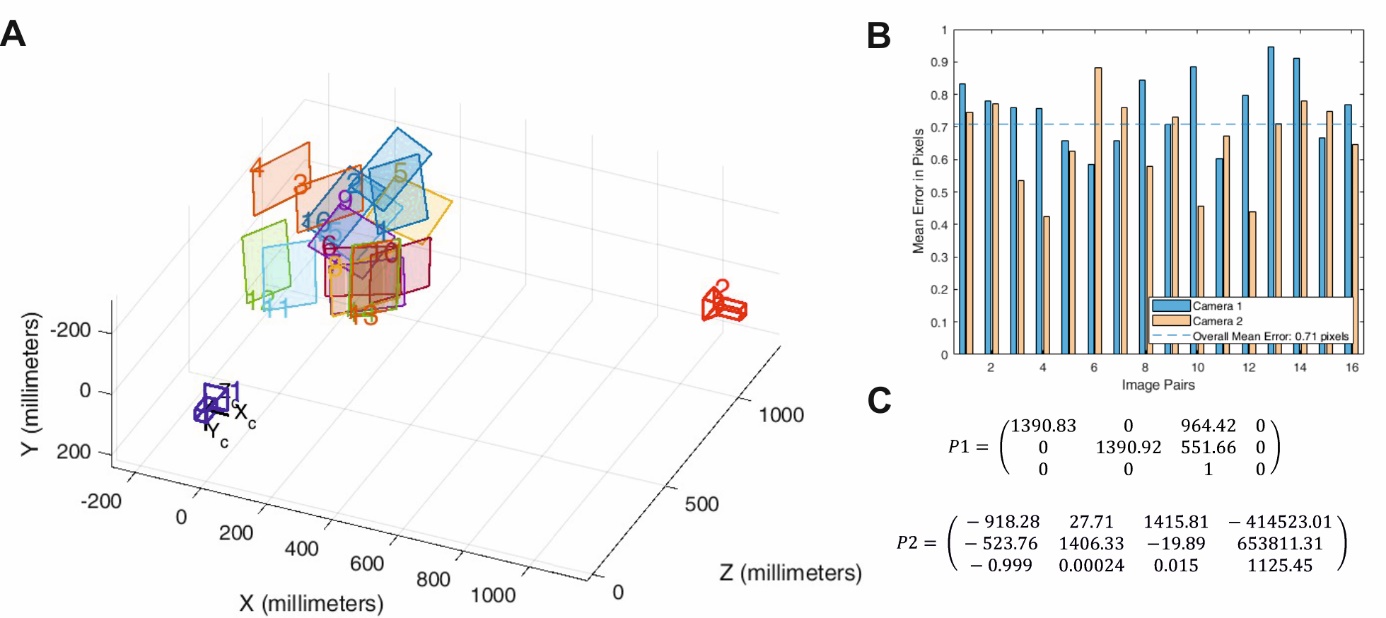
Figure S1: Camera Parameters.** Orientation of cameras and schematic representations of the locations of calibration boards (A) and mean reprojection errors of calibration images (B) both generated with the Stereo Camera Calibration Toolbox (Matlab 2022b; The MathWorks Inc., USA). The calculated projection matrices (C) with Camera 1 (purple camera, a) being the center of the reconstructed scene.


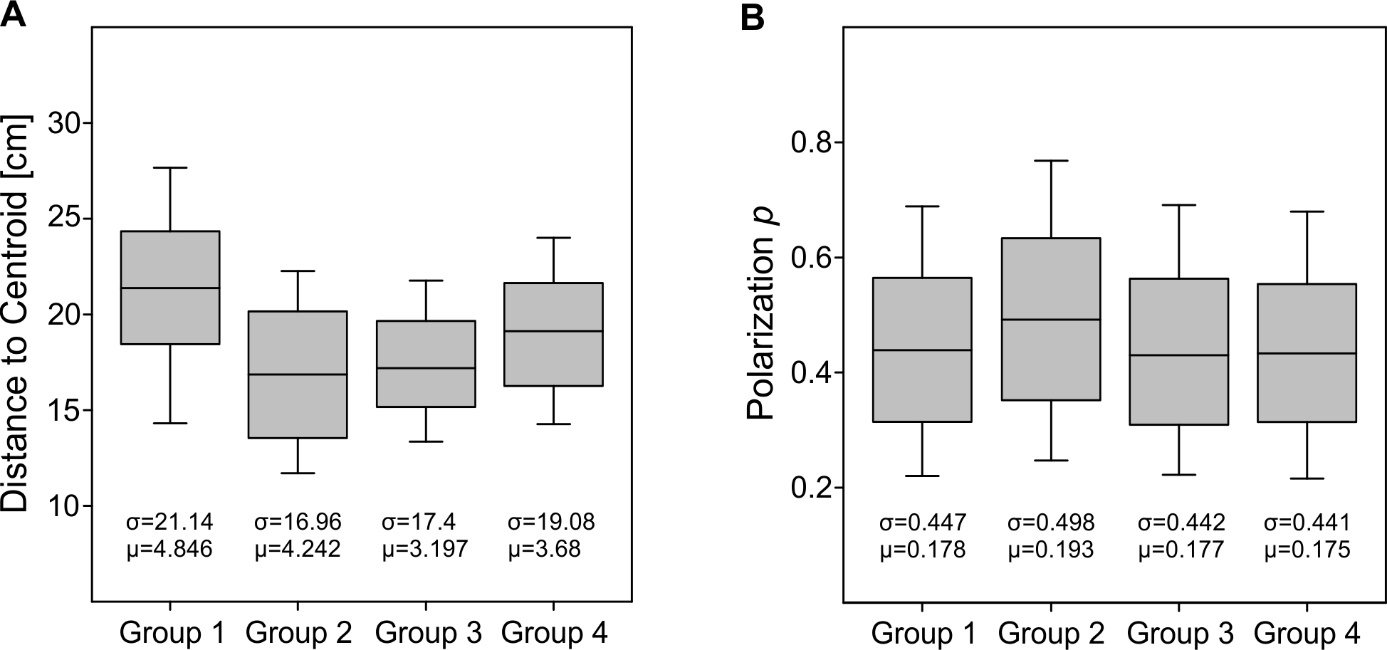


**Figure S2: Parameters for the description of group behaviors in shoals of *A. katoptron*.** In our study, four groups each consisting of five individuals were tested. To describe the shoaling behavior, we determined the mean distance to the group’s centroid (A) and the polarization (B), a measure of alignment the groups individuals. Values *p* = 1 indicate the maximum alignment of all members of the group. Below the boxplot, mean (*σ*) and standard deviation (*μ*) are shown.

Figures were created with SigmaPlot 12.0 and processed with CorelDraw Graphics Suite 2017.

**
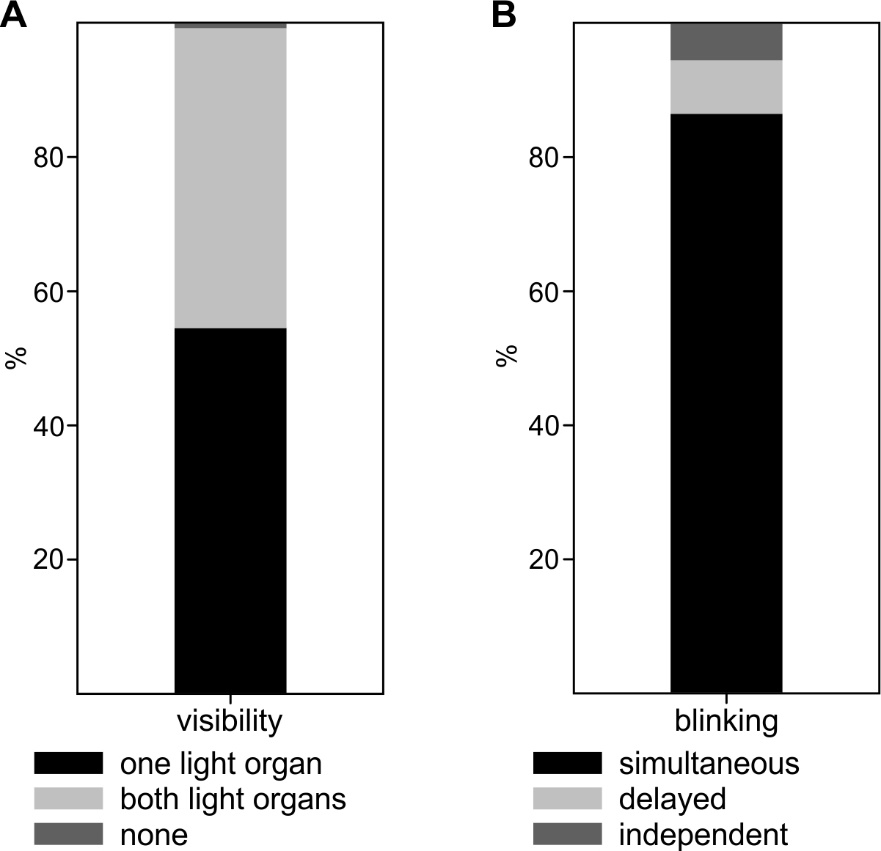
**

**Figure S3: Estimation of visibility (A) and simultaneous blinking (B) of light organs.** For each fish (*n*=20), we analyzed for one minute if either none, one or both light organs were simultaneously observable in the camera perspectives (A). In addition, we analyzed if both light organs were simultaneously exposed/occluded, a delay between both light organs occurred during light organ exposure or were exposed independently (B). Delayed blinking occurred when one light organ was unequally exposed in a simultaneous blink event. Blinking was considered independent when both light organs were closed before and after the exposure of only one light organ.

Figures were created with SigmaPlot 12.0 and processed with CorelDraw Graphics Suite 2017.

**Table S1: Statistical analysis of turning angle.** Shown are the results for the two-way rm‑ANOVA with Holm-Sidak post-hoc analysis (see Figure 3E). We analyzed turning angle at the six timesteps (three before and after light organ transition). We used timestep and type of transition (either exposed to occluded light organs or vice versa) as factors. Statistical significance was calculated in SigmaPlot 12.0.

**Normality Test (Shapiro-Wilk)** Passed (P = 0.415)

**Equal Variance Test:** Passed (P = 0.213)

**Source of Variation DF SS MS F P**

Individual 19 6007.952 316.208

Switch 1 1491.361 1491.361 184.70 <0.001

Switch x Individual 19 153.773 8.093

Frame 5 1584.837 316.967 50.834 <0.001

Frame x Individual 95 592.361 6.235

Switch x Frame 5 3528.925 705.785 63.613 <0.001

Residual 95 1054.027 11.095

Total 239 14413.236 60.306

The effect of different levels of Switch depends on what level of Frame is present. There is a statistically significant interaction between Switch and Frame. (P = <0.001)

All Pairwise Multiple Comparison Procedures (Holm-Sidak method): Overall significance level = 0.05

Comparisons for factor: **Switch within 1**

**Comparison Diff of Means t P P<0.05**

2 vs. 1 6.815 6.621 <0.001 Yes

Comparisons for factor: **Switch within 2**

**Comparison Diff of Means t P P<0.05**

2 vs. 1 1.624 1.578 0.117 No

Comparisons for factor: **Switch within 3**

**Comparison Diff of Means t P P<0.05**

1 vs. 2 12.540 12.183 <0.001 Yes

Comparisons for factor: **Switch within 4**

**Comparison Diff of Means t P P<0.05**

1 vs. 2 14.867 14.444 <0.001 Yes

Comparisons for factor: **Switch within 5**

**Comparison Diff of Means t P P<0.05**

1 vs. 2 8.189 7.956 <0.001 Yes

Comparisons for factor: **Switch within 6**

**Comparison Diff of Means t P P<0.05**

1 vs. 2 2.757 2.678 0.009 Yes

**Table S2: Statistical analysis of swimming speed.** Shown are the results for the two-way rm‑ANOVA with Holm-Sidak post-hoc analysis (see Figure 3F). We analyzed swimming speeds at the six timesteps (three before and after light organ transition). We used timestep and type of transition (either exposed to occluded light organs or vice versa) as factors. Statistical significance was calculated in SigmaPlot 12.0.

**Normality Test (Shapiro-Wilk)** Passed (P = 0.105)

**Equal Variance Test:** Passed (P = 0.356)

**Source of Variation DF SS MS F P**

Individual 19 0.628 0.033

Switch 1 0.053 0.053 143.075 <0.001

Switch x Individual 19 0.007 0.0003

Frame 5 0.047 0.0094 41.917 <0.001

Frame x Individual 95 0.021 0.0002

Switch x Frame 5 0.182 0.0364 38.719 <0.001

Residual 95 0.089 0.0009

Total 239 1.029 0.0043

The effect of different levels of Switch depends on what level of Frame is present. There is a statistically significant interaction between Switch and Frame. (P = <0.001)

All Pairwise Multiple Comparison Procedures (Holm-Sidak method): Overall significance level = 0.05

Comparisons for factor: **Switch within 1**

**Comparison Diff of Means t P P<0.05**

2 vs. 1 0.0643 6.993 <0.001 Yes

Comparisons for factor: **Switch within 2**

**Comparison Diff of Means t P P<0.05**

2 vs. 1 0.0862 9.372 <0.001 Yes

Comparisons for factor: **Switch within 3**

**Comparison Diff of Means t P P<0.05**

2 vs. 1 0.0885 9.621 <0.001 Yes

Comparisons for factor: **Switch within 4**

**Comparison Diff of Means t P P<0.05**

2 vs. 1 0.0238 2.588 0.011 Yes

Comparisons for factor: **Switch within 5**

**Comparison Diff of Means t P P<0.05**

1 vs. 2 0.0377 4.103 <0.001 Yes

Comparisons for factor: **Switch within 6**

**Comparison Diff of Means t P P<0.05**

1 vs. 2 0.0463 5.038 <0.001 Yes

**Additional File 2**

Additional File 2_Raw_Data.xlsx

The spreadsheet contains all data points necessary to interpret or reanalyze our results. It includes individual tracking profiles and gives the data points used for statistical analysis.

**Additional File 3**

Additional File 3.mp4

The video shows shoaling behavior of a group of flashlight fish *Anomalops katoptron* in the laboratory. Both camera perspectives, that were necessary for our analysis, are included.
